# Urine-Derived Cells in Kidney Transplantation: Linking Cellular Phenotypes, Secretome Signatures and Multi-Omic Technologies

**DOI:** 10.3390/cells15171622

**Published:** 2026-09-07

**Authors:** Valeria Pizzuti, Marcello Demetri, Emma Balducelli, Sofia Bin, Michela Verasani, Elisa Gessaroli, Valeria Corradetti, Gaetano La Manna, Giorgia Comai

**Affiliations:** 1 Nephrology, Dialysis and Kidney Transplant Unit, IRCCS Azienda Ospedaliero-Universitaria di Bologna, Via Giuseppe Massarenti 9, 40138 Bologna, Italy; valeria.pizzuti@aosp.bo.it (V.P.); marcello.demetri@aosp.bo.it (M.D.); valeria.corradetti@aosp.bo.it (V.C.); giorgia.comai2@unibo.bo.it (G.C.); 2 Department of Medical and Surgical Sciences (DIMEC), Alma Mater Studiorum-University of Bologna, 40126 Bologna, Italy; emma.balducelli2@unibo.it (E.B.); sofia.bin2@unibo.it (S.B.); michela.verasani2@unibo.it (M.V.); elisa.gessaroli5@unibo.it (E.G.)

**Keywords:** kidney transplant, urine-derived cells, secretome, extracellular vesicles, multi-omics

## Abstract

Kidney transplant monitoring and early identification of graft dysfunction are central needs for long-term graft survival. Although kidney biopsy represents the gold standard in diagnostic procedures, its invasiveness may limit frequent longitudinal evaluation, highlighting the necessity of non-invasive diagnostic tools. Urine has emerged over the years as an easily obtainable source of cellular and molecular components derived from transplanted kidneys. Particularly, exfoliated cells and extracellular vesicles (EVs) represent complementary and interconnected markers able to reflect tissue injury, inflammatory signals, immune cell infiltration, and regenerative processes occurring in the graft. Also, recent advances in transcriptomics, proteomics, metabolomics, and single-cell technologies have expanded the diagnostic and mechanistic value of urinary liquid biopsy, enabling the identification of disease-specific molecular signatures associated with rejection, delayed graft function, fibrosis, and graft loss. This review discusses the emerging concept of an integrated urinary cell–EV ecosystem and highlights how integrated multi-omic approaches may transform non-invasive graft surveillance and advance precision medicine in kidney transplantation.

## 1. Introduction

Urine represents a unique and non-invasive source of cellular and molecular information reflecting physiological and pathological processes occurring throughout the nephrons and urinary tract. In recent years, increasing attention has been directed toward urinary exfoliated cells and extracellular vesicles (EVs) as dynamic components of a complex liquid biopsy ecosystem capable of mirroring renal injury, inflammation, fibrosis, immune activation, and tissue repair. Although tissue biopsy is considered the gold standard in monitoring the graft during kidney transplant follow-up, the search for urine-derived components able to reflect the status of the organ could overcome the risks and limitations associated with repeated allograft biopsies, which are characterized by invasiveness of the procedure, bleeding and infections, psychological impact on the patient, and high costs of sample processing [1].

**Figure 1:**
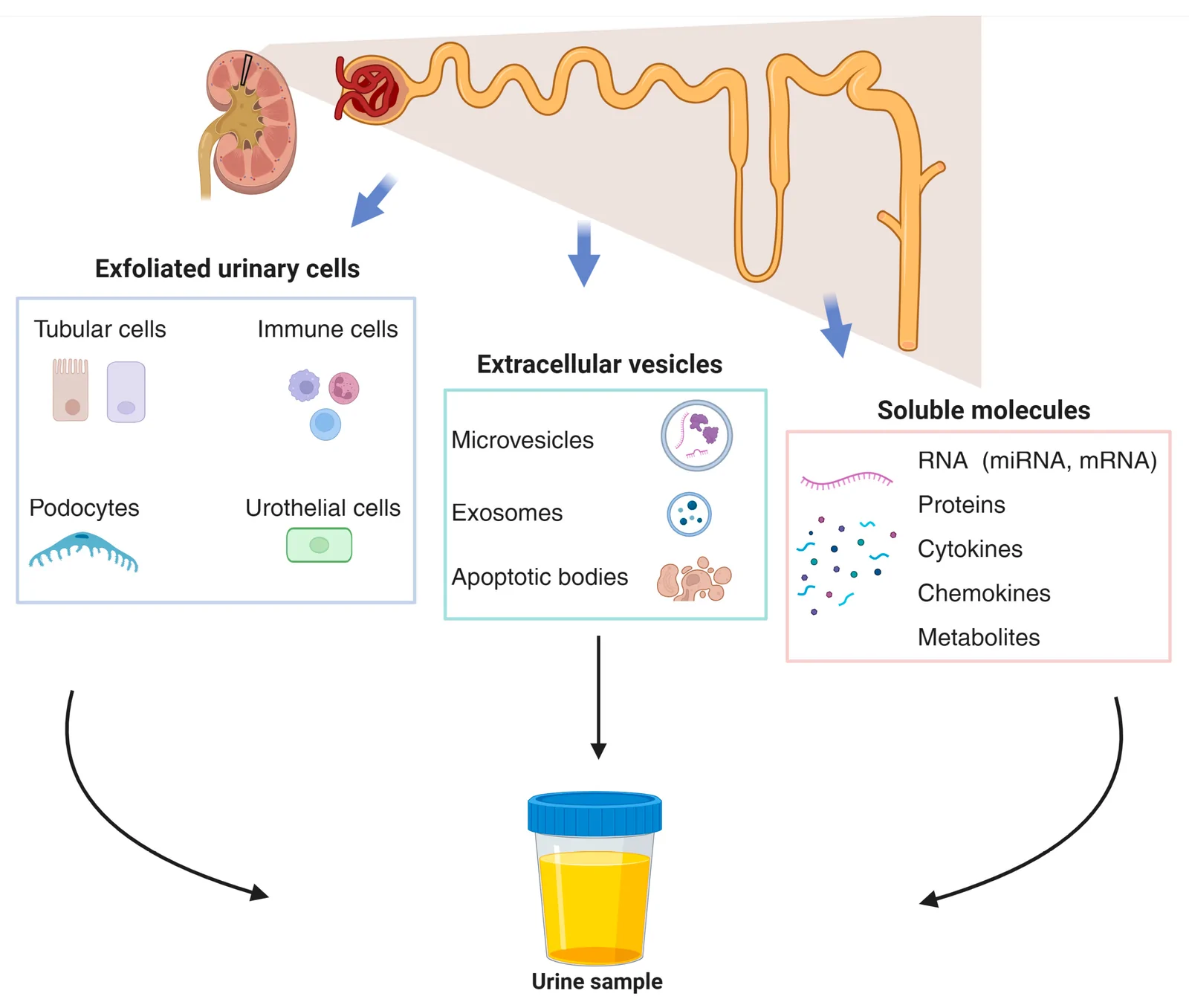
Main components of the urinary liquid biopsy. Schematic representation of the principal biomarker sources in urine, including exfoliated urinary cells, extracellular vesicles, and soluble molecules released from the kidney and urinary tract. Created in BioRender. Bin, S. (2026), https://BioRender.com/lyvl83k, accessed on 4 September 2026.

Beyond their potential diagnostic role, growing evidence suggests that urinary cellular and secretomic profiles may provide mechanistic insights into disease progression and may contribute to the development of regenerative and cell-free therapeutic strategies. Figure 1 describes the main kidney-derived components that could be find in urine.

This review aims to provide a comprehensive overview of urinary exfoliated cells in kidney transplant recipients and to explore how cellular phenotypes, secretome-derived signals, and multi-omics technologies can be integrated to improve the non-invasive assessment of allograft status. By linking cellular composition, functional activity, and molecular signatures, we discuss the potential of urinary cell-based liquid biopsy as a next-generation platform for the early detection of rejection, graft injury, and long-term transplant dysfunction.

## 2. Literary Search Strategy

This narrative review was conducted through a literature search performed on PubMed, Scopus, Web of Science, and other relevant reference lists up to June 2026. The search combined the following keywords and Boolean operators: “kidney transplantation”, “urine-derived cells”, “urinary biomarkers”, “extracellular vesicles”, “secretome”, “transcriptomics”, “proteomics”, “metabolomics”, “liquid biopsy”, and “multi-omics”. Priority was given to original studies, systematic reviews, and recent evidence addressing the biological basis, methodological aspects, and clinical applications of urinary biomarkers in kidney transplantation. Additional relevant publications were identified through manual screening of reference lists. No formal systematic review protocol or meta-analysis methodology was applied. Table 1 summarizes the main findings of the cited studies.

## 3. Urinary Cellular Compartment

### 3.1. Biological Origin of Urinary Cells

The kidney and urinary tract are composed of highly specialized cellular populations that differ in morphology, anatomical localization, and physiological function. The isolation and characterization of these cell types have broad applications in diagnostics, disease modeling, drug screening, basic research, and regenerative medicine. Traditionally, the acquisition of renal cells requires tissue biopsies, which are invasive procedures associated with potential complications and patient discomfort. Furthermore, the isolation of viable cells from solid tissues generally relies on mechanical and enzymatic dissociation protocols that may reduce cell yield, viability, and proliferative capacity [25]. Therefore, non-invasive procedures are highly desirable to increase the viability and growth of primary cells. In this context, urine meets these needs, since it is an easily obtainable cell source from which it is possible to isolate cells without ethical repercussions and risk for the patients. Cell exfoliation is a physiological mechanism that contributes to tissue homeostasis by regulating epithelial cell turnover and maintaining the integrity of the nephron. This process involves the progressive detachment of cells from the extracellular matrix, ultimately leading to anoikis, a form of programmed cell death triggered by the loss of cell–matrix interactions. In kidney transplantation, epithelial detachment is markedly enhanced by ischemia–reperfusion injury, alloimmune-mediated damage, calcineurin inhibitor toxicity, and chronic inflammatory processes. Excessive anoikis contributes to tubular epithelial cell depletion and impairment of the epithelial barrier, and it may promote maladaptive repair, ultimately favoring interstitial fibrosis and tubular atrophy (IFTA), one of the major pathological determinants of chronic graft dysfunction [26]. Conversely, activation of autophagy has been shown to protect detached epithelial cells from anoikis by preserving cellular homeostasis and promoting survival under stress conditions. In experimental models of kidney injury, autophagy also limits oxidative stress, mitochondrial dysfunction, and inflammatory signaling, thereby attenuating tubular injury and fibrogenesis. Consequently, the balance between anoikis and autophagy may influence both the composition of urinary exfoliated cells and the progression of graft injury, making these pathways biologically relevant for the interpretation of urinary liquid biopsy findings [27,28].

Exfoliated cells are released into several biological fluids, including breast milk, gastrointestinal secretions, and urine. Although the majority of these cells exhibit an epithelial phenotype, the exfoliated cell compartment is highly heterogeneous and includes subpopulations that retain clonogenic and proliferative capabilities. Urinary cell composition includes tubular epithelial cells originating from different nephron segments, podocytes, parietal epithelial cells, urothelial cells, inflammatory cells, and rare progenitor-like populations. Importantly, the relative abundance of these populations changes substantially according to the physiological or pathological condition of the kidney [29]. In healthy individuals, urinary cells are predominantly terminally differentiated epithelial cells with limited proliferative capacity. Conversely, kidney injury promotes increased shedding of viable epithelial and inflammatory cells that retain the molecular characteristics of their tissue of origin, making urine an attractive source of non-invasive biomarkers [30,31]. Changes in the urinary cellular profile have been described in acute kidney injury (AKI), diabetic nephropathy, ischemia–reperfusion injury, glomerular diseases, and chronic kidney disease. These observations have stimulated growing interest in the use of urinary cell signatures as non-invasive biomarkers for disease diagnosis, prognosis, and therapeutic monitoring [29,32].

### 3.2. Urinary Exfoliated Cells in Kidney Transplantation

Kidney transplant is considered the preferred kidney replacement therapy for eligible patients and represents the treatment of choice for end-stage kidney disease (ESKD), offering superior survival and quality of life compared with dialysis. Despite the advancement in immunosuppressive therapies, surgical procedures and donor management, kidney recipients are at risk of several complications, including rejection, delayed graft function (DGF), drug toxicity and infections [31]. Thus, graft monitoring during transplant follow-up is essential, and kidney biopsies are the most effective tools for the assessment of graft disorders [33,34]. Given the invasiveness of the procedure, the risk of side effects and the higher costs, non-invasive approaches for transplant monitoring have been investigated over the years, including the analysis of blood and urine. Among the different liquid biopsy approaches investigated to date, urine represents a particularly attractive source of biological material because of its accessibility, repeatability, and direct contact with the transplanted kidney [35,36]. Urine contains a heterogeneous population of both donor and recipient-derived cells; these cells retain morphological, phenotypic, and molecular characteristics of their tissue of origin, thereby providing a unique opportunity to investigate ongoing biological processes within the allograft. In addition to cellular profiling, urinary liquid biopsy approaches allow the simultaneous analysis of EVs, proteins, cytokines, chemokines, growth factors, and nucleic acids, offering a comprehensive overview of graft health [37]. The flow cytometry characterization of exfoliated cells derived from transplanted patients revealed that most of the cells have an epithelial phenotype, expressing keratins as Keratin 8 (KRT8) and KRT18 [38]. Several studies have further shown that these cells predominantly originate from the upper urinary tract and preserve segment-specific molecular signatures corresponding to their nephron compartment of origin. In a study conducted by our group on a cohort of kidney transplant patients, we confirmed the high cytokeratin expression and the presence of other tubular markers such as CD13 and EpCAM. Also, a subset of exfoliated cells expressed both the CD24 and CD133 surface markers [2]. The coexpression of these markers characterizes the kidney epithelial progenitor cells, which play a pivotal role in kidney regeneration [37,38]. Previous studies also demonstrated that progenitor cells derived from urine showed similar phenotypical and functional features to cells derived from kidney biopsies [39,40].

The detection of specific urinary cell populations has also been associated with clinically relevant outcomes. In particular, the presence of urinary parietal epithelial cells (uPECs) has emerged as a potential indicator of glomerular injury and adverse graft evolution. Manonelles and colleagues demonstrated that kidney transplant recipients with detectable uPECs six months after transplantation exhibited increased podocyturia and a higher proportion of proliferating parietal epithelial cells within protocol biopsies. Although no significant differences in baseline clinical parameters were observed between patients with or without urinary PECs, the presence of uPECs was associated with progressive deterioration of renal function, increased proteinuria, and more pronounced chronic allograft damage during long-term follow-up [3]. These results suggest that urinary PEC detection may reflect ongoing glomerular remodeling processes and identify patients at increased risk of chronic graft dysfunction. However, these findings derive from a relatively small single-center cohort, and urinary contamination as well as incomplete availability of follow-up protocol biopsies represent relevant limitations; therefore, the prognostic value of uPECs requires independent validation.

Goerlich and colleagues aimed to establish a non-invasive diagnostic tool to monitor kidney transplant patients by flow cytometry analysis of urine samples, focusing on both immune cell subsets and kidney-derived cells. They observed that patients without rejection had a higher percentage of tubular cells and podocalyxin (PDX)-positive cells, with a decrease in the T cell populations compared with patients with rejection. While the urinary increase in the T cell subset in the presence of rejection reflects the intrarenal inflammation signature, the number of tubular cells and PDX-positive cells was not related to histological findings [4]. Indeed, the unexpectedly higher abundance of tubular and PDX-positive cells in patients without rejection contrasts with their proposed interpretation as direct markers of tissue damage. Moreover, PDX is not exclusively expressed by podocytes, further limiting the cellular specificity of this marker. Urinary T cells alone also failed to clearly discriminate rejection from other causes of graft dysfunction, supporting the use of combined rather than single-cell signatures.

In a recent study, Wu and colleagues aimed to analyze the profile of exfoliated proximal tubular cells (PTCs) obtained from kidney transplant patients to evaluate the correlation between urinary cell signature and graft dysfunction, in particular acute tubular necrosis (ATN), graft rejection or non-rejection-associated IFTA. They first isolated cells by using anti-CD13 and anti-sodium-glucose co-transport 2 antibodies immunomagnetic separation and evaluated the multispectral autofluorescence signals through machine learning analysis. Despite the limitations of the study, including the small sample size and its retrospective nature, results showed different multispectral autofluorescence signals among the tested groups, presenting a promising proof of concept study regarding the potential application of exfoliated cells in discriminating patients with various histopathologic complications [5]. These findings demonstrate that urinary exfoliated cells represent a clinically relevant component of the urinary liquid biopsy, reflecting the ongoing processes occurring within the transplanted kidney such as epithelial injury, immune activation, regenerative responses, and chronic remodeling. The assessment of the urinary cell profile may become a useful tool for the non-invasive monitoring of kidney transplant patients and for the early diagnosis of graft dysfunctions. Nevertheless, the interpretation of urinary cellular profiles requires caution. Contrary to common assumptions, urinary cells cannot be considered exclusively donor-derived. While tubular epithelial cells generally originate from the transplanted kidney, inflammatory leukocytes are predominantly recipient-derived. Moreover, urothelial cells may be derived from the bladder or urethra, particularly in the presence of urinary tract inflammation or instrumentation. Consequently, the tissue origin of urinary cells should always be interpreted according to the experimental methodology adopted in each study, including lineage-specific markers, donor–recipient genetic discrimination, or sex chromosome analysis whenever available [41].

### 3.3. In Vitro Culture and Characterization of Urine-Derived Cells

It is well known that in vitro culture methods induce a selection of cell populations and may alter their phenotypical and functional features. Also, long-term expansion promotes the onset of early senescence. However, the isolation and culture of primary cells represents an important tool to investigate their potential applications, their response to the surrounding microenvironment, and their interaction with other cell populations. Accordingly, findings obtained from expanded urinary cells should not be directly extrapolated to the composition or biological behavior of freshly exfoliated cells in vivo.

Longitudinal analyses performed by our group on transplant recipients revealed dynamic changes in the urinary cellular compartment during post-transplant follow-up. At early time points after transplantation, urinary cells from nearly all patients exhibited robust proliferative capacity and could be successfully expanded in vitro. However, both cell isolation efficiency and proliferative potential progressively declined at later follow-up intervals, including six months and one year after transplantation. For what concerns the features of cultured URECs, these cells showed promising immunomodulatory properties when cultured with Peripheral Blood Mononuclear Cells (PBMCs), inducing a reduction in CD4 and CD8 T lymphocyte proliferation and an increase in the T regulatory cell subset. Interestingly, in vitro treatment of urine-derived cells with neutrophil gelatinase-associated lipocalin (NGAL), a well-established marker of kidney injury, significantly impaired their immunomodulatory functions, suggesting that the exposure to damage-related markers may alter the biological properties of urine-derived progenitor cells [6]. However, the pathways behind the immunomodulatory properties of URECs and the inhibitory effect of NGAL still require investigation. Moreover, these observations derive from in vitro experimental systems, and their relevance to the immunological environment of the transplanted kidney remains to be established. Knafl and colleagues investigated the in vitro detection of tubular cell agglomerates known as nephrospheres in 45 kidney-transplanted and 19 non-transplanted individuals [7]. Results revealed that nephrospheres were not detected in non-transplanted individuals, while their presence was observed in 17 kidney-transplant recipients one week after onset of AKI and were detectable until kidney function was recovered, suggesting their potential use in evaluating recovery from AKI.

However, daily follow-up was not obtained from all patients enrolled, and molecular analysis was not performed specifically on nephrospheres but on the whole urinary sediment, suggesting the need for a better characterization of these cell structures and their diagnostic use. Furthermore, although nephrosphere detection showed high specificity for recovery, its sensitivity was only approximately 61%, indicating that their absence cannot reliably identify patients who will fail to recover. The absence of nephrospheres in non-transplanted AKI patients also remains mechanistically unexplained, limiting the generalizability of this finding beyond the transplant setting. Further in vitro studies on exfoliated cells demonstrated that the urine-derived progenitor subset retains clonogenic and amplification potential, allowing the establishment of cell culture and expansion under proper culture conditions [42]. It is well recognized that the culture medium composition strongly influences the growth of specific cell types after urine sediment seeding; indeed, epithelial cells, stromal cells, and immune cells require different culture conditions. The choice of proper culture conditions is a relevant aspect to consider in the setup of the study of a urinary derived population and their phenotypic and secretory signatures, representing an important source of methodological bias, since culture itself selects specific cellular populations and may modify the phenotype observed after expansion [43]. Overall, current evidence supports urinary exfoliated cells as a promising research and monitoring tool, but differences between freshly isolated and cultured cells, limited cohort sizes, methodological heterogeneity, and the lack of multicenter prospective validation currently prevent their routine use as standalone biomarkers in kidney transplantation.

## 4. Urinary Secretome in Kidney Transplantation

### 4.1. Soluble Urinary Secretome

The urinary secretome comprises the entire repertoire of soluble and vesicle-associated molecules released by renal resident cells, infiltrating immune cells, endothelial cells, and cells lining the urinary tract. These molecules are continuously secreted under physiological conditions to maintain tissue homeostasis and intercellular communication, while their composition dynamically changes during inflammation, ischemia–reperfusion injury, immune activation, fibrosis, and tissue repair [44].

Consequently, the urinary secretome represents one of the most comprehensive sources of biological information available through non-invasive liquid biopsy. From a biological perspective, the urinary secretome consists of two complementary components: the soluble secretome, including cytokines, chemokines, growth factors, complement proteins, enzymes, metabolites, and soluble receptors freely dissolved in urine, and the vesicular secretome, represented by EVs carrying proteins, lipids, messenger ribonucleic acids (RNAs), microRNAs, long non-coding RNAs, circular RNAs, and other bioactive molecules protected by a lipid bilayer. Although these two compartments are often investigated separately, they should be regarded as complementary mediators of the same biological processes occurring within the kidney allograft. Several soluble urinary biomarkers have already demonstrated diagnostic or prognostic potential in kidney transplantation [45]. Among them, NGAL and Kidney Injury Molecule-1 (KIM-1) have been extensively investigated as early indicators of tubular injury, since their increase in urine occurs before the serum creatinine increase. In a longitudinal study involving 70 transplanted patients, KIM-1 and NGAL were tested as post-transplant renal function prognostic biomarkers. Although the reduced availability of kidney biopsies limited the possibility of properly correlating the biomarkers and the anatomopathological findings, researchers observed that NGAL values were associated with a higher probability of DGF onset, consistent with previous findings [46,47].

In contrast, in other studies, no significant correlations have emerged among urinary KIM-1 increase and graft complications, requiring further investigation [45,48]. The quantification of urinary chemokines during graft monitoring has been validated in several studies. Particularly, urinary chemokine (C-X-C motif) ligand (CXCL)9 and CXCL10 have emerged as robust indicators of inflammation and cellular infiltration, with a high negative predictive value (NPV) for the diagnosis of acute rejection (AR) [49,50]. Additional molecules, including monocyte chemoattractant protein-1 (MCP-1), osteopontin, interleukin-18 (IL-18), transforming growth factor-β (TGF-β), and components of the complement cascade, have also been associated with inflammation, fibrosis, and chronic allograft dysfunction [51]. Despite the encouraging findings that have emerged over the years, the clinical interpretation of urinary biomarkers remains challenging. The concentrations of soluble molecules may be influenced by urinary dilution, proteinuria, urinary tract infections, ischemic injury, and concomitant inflammatory conditions. Consequently, no single soluble biomarker currently provides sufficient diagnostic accuracy to replace kidney biopsy. Instead, soluble biomarkers should be considered complementary tools that contribute to a multidimensional assessment of graft status when integrated with cellular, vesicular, and molecular information [52].

### 4.2. Urinary EVs

According to the minimal information for studies of extracellular vesicles (MISEV2023) guidelines, EVs are cell-released particles delimited by a lipid bilayer that are incapable of self-replication because they lack a functional nucleus. Unless a specific biogenesis pathway has been experimentally demonstrated, the generic term “EVs” is recommended in preference to terms such as “exosomes” or “microvesicles”. These particles are produced by different cell types and secreted in almost every body fluid [53]. EVs contribute to the communication among cells and tissues, carrying proteins, lipids, metabolites, messenger RNAs, microRNAs, and other bioactive molecules. Over the years, EVs have emerged as key regulators of tissue homeostasis, immune responses, injury repair, and regeneration [54].

Within the kidney, EVs mediate intercellular communication among different nephron segments, involving intraglomerular, glomerular–tubular, and tubular–interstitial communication. Depending on the cellular origin, molecular cargo, and surrounding microenvironment, EV-related networks may exert protective effects, contributing to renal homeostasis, immunomodulation and repair, or, conversely, promote fibrosis and inflammation [8,55]. Their role as carriers of both repair and immunomodulatory signals, as well as proinflammatory or profibrotic messages, make EVs a reliable tool in the investigation of pathophysiological processes occurring along the urinary tract [55]. uEVs have been extensively characterized in various kidney-related pathologies, since they retain signatures characteristic of several segments of the nephron, reflecting the physiological and pathological state of the organ. Moreover, their structure protects the molecular cargo from degradation, providing a stable and reliable source of information. Several studies demonstrated that uEVs are predominantly released by epithelial cells lining the first segment of nephrons, with limited contribution of the lower urinary tract segment and a low presence of plasma-derived EVs [56,57].

### 4.3. Urinary Extracellular Vesicles as Biomarkers of Allograft Injury

Given the already mentioned characteristics of uEVs, several studies focused on their possible application as non-invasive biomarkers in kidney transplant management and graft function evaluation [37,58]. It has been demonstrated that specific EV subsets are associated with AR. A urine-based device was recently developed to detect kidney transplant rejection by analyzing EVs released from immune cells.

Basing on their results, higher levels of CD3^+^ EVs were found in transplant patients undergoing T cell-mediated rejection (TCMR), supporting the potential use of these particles in mirroring intrarenal immune response [59]. Recent evidence has further expanded the spectrum of EV-based biomarkers in kidney transplantation. In addition to immune cell-derived EVs, reduced levels of plasma EVs derived from B cells, T cells, and endothelial cells have been associated with antibody-mediated rejection (AMR), whereas lower urinary endothelial-derived EVs have been reported in patients with TCMR. These findings further support the concept that EV phenotyping may provide clinically relevant information on the immunological status of the graft [9].

The diagnostic potential of urinary vesicles has been further expanded owing to the advancement in proteomic analyses. Proteins packaged within EVs more likely reflect the physiological and pathological processes occurring within the urinary tract. Consequently, EV proteomics has emerged as a promising approach for identifying disease-specific molecular signatures associated with graft injury. A study involving patients with normal kidney function, end-stage renal disease, or kidney transplantation aimed to compare the proteomic profile of uEVs in the three different groups. Based on high-resolution mass spectrometry results, end-stage renal disease was associated with an alteration in energy metabolism, cytoskeleton organization and cell motility, while transplant patients showed proteomic alterations mostly related to the fibrotic process [10,11].

Interestingly, proteins such as cystatins and gelsolin detected in uEVs have emerged as candidates for kidney transplant monitoring. While the first protein family is considered an indicator of graft function, gelsolin has been mainly associated with podocyte damage and chronic kidney disease progression. However, these findings were primarily derived from exploratory proteomic analyses, and the identified signatures have not yet been extensively validated in independent transplant cohorts or demonstrated to be specific for rejection rather than reflecting kidney dysfunction or injury more broadly.

Additional evidence has been obtained from a cohort of pediatric patients receiving kidney transplant. When comparing patients with AR and patients with stable graft function, it has been observed that the numbers of uEVs did not significantly differ among the two groups. Conversely, the EV proteomic profile was found highly informative, showing a specific pattern in patients undergoing rejection. These findings strengthen the importance of EV composition analysis and cargo evaluation rather than relying only on quantitative assessment [12]. These results derive from a small, single-center pilot cohort, with only a limited number of rejection events. Moreover, the limited representation of specific rejection phenotypes, including AMR, and the absence of systematic surveillance biopsies may have affected patient classification and limited the generalizability of the identified proteomic signature. Despite the limitations of most of the studies, commonly due to the small sample size, the study design and the risk of selection bias, these results indicate that urinary vesicles are a reliable tool reflecting the degree of renal dysfunction, and the alteration in EV signature could anticipate the common parameters used to assess kidney function. The EV profile could also significantly contribute to the understanding of reparative and regenerative mechanisms occurring in the graft as well as the recognition of damage-associated pathways [55]. The role of kidney progenitor cells in maintaining homeostasis and their contribution to tissue regeneration has been investigated by the assessment of urinary EVs expressing progenitor markers and their correlation with clinical outcomes. When referring to kidney transplant recipients, urinary CD133^+^ EVs were detected at lower levels in patients showing a slow graft function recovery, with an increase after seven days from transplantation. The higher presence of CD133^+^ uEVs in patients with normal graft function recovery, along with their absence in patients with ESKD, suggest that the quantification and characterization of urinary EVs may be indicators of the regeneration processes and functional recovery occurring in the graft [55].

Nevertheless, these findings should be considered preliminary, as the biological interpretation of progenitor-derived EVs remains complex, and their relationship with tissue regeneration does not necessarily establish a direct mechanistic or prognostic role. In a longitudinal analysis of serum and urine EV profiles on a selected cohort of transplant patients, an EV signature was associated with post-transplant outcome. Focusing on uEV findings, the authors observed that the progressive increase in CD1c, CD105, CD133 and SSEA-4 markers in transplant patients was associated with the recovery of renal function during follow-up, highlighting the importance of the regenerative processes in graft function resumption and the potential application of EVs in kidney transplant monitoring [8].

The combined analysis of exfoliated cells and EVs in the liquid biopsy process offers a complementary insight of both the secretome profile and signaling pathways as well as the cell turnover and exfoliation processes, improving the development of disease-specific urinary patterns. This perspective could be further implemented with the parallel assessment of damage-associated markers [60]. Referring to NGAL measurement, it has been observed that the urinary amount of this early biomarker of kidney damage was higher in the EV subset compared with the cellular fraction in kidney transplant patients. Another relevant result was related to the DGF and no DGF classification of patients; while NGAL content was similar in the cellular fraction, its expression was higher in urinary EVs derived from DGF patients, correlating with serum creatinine [61,62].

### 4.4. Methodological Challenges and Standardization of Urinary Extracellular Vesicle Analysis

Urinary physicochemical properties, including hydration status, pH, osmolality, and protein concentration, vary considerably both within and between individuals. Consequently, pre-analytical variables such as the timing of urine collection, processing delays, centrifugation protocols, storage conditions, and repeated freeze–thaw cycles introduce technical variability that may alter EV yield and obscure biologically relevant differences.

Thus, the translation of uEVs into routine clinical practice remains hampered by substantial methodological heterogeneity [53]. The ISEV Urine Task Force further emphasizes that no isolation technique is universally optimal for urinary EV analysis; widely adopted methods—including differential ultracentrifugation, size-exclusion chromatography, ultrafiltration, polymer-based precipitation, and immunoaffinity capture—differ substantially in terms of recovery, purity, scalability, and compatibility with downstream analyses [63]. As a result, differences in isolation methodology frequently translate into divergent proteomic, transcriptomic, and metabolomic profiles, complicating direct comparisons across studies and limiting reproducibility. A unique challenge in urinary EV research is represented by uromodulin (Tamm–Horsfall protein), the most abundant protein in healthy urine [64].

The uromodulin polymeric filamentous network traps EVs, thereby reducing their recovery and introducing contaminants that interfere with their quantification and characterization. Strategies designed to dissociate or remove uromodulin can improve EV recovery, although there is no consensus on the best methods, and effectiveness varies according to the isolation procedure [65]. Another unresolved issue concerns urinary concentration, which varies substantially among individuals and over time. Several data normalization strategies have been proposed, including adjustment for urinary creatinine, urine volume, total protein concentration, particle number, or EV-specific markers [66,67]. However, as demonstrated in the position paper of Erdbrügger and colleagues, each approach corrects for different sources of variability, and none has emerged as universally applicable across experimental settings [63].

Collectively, these methodological limitations represent one of the principal barriers to the clinical implementation of urinary EV biomarkers. Continued efforts toward protocol harmonization, rigorous reporting in accordance with the MISEV recommendations, and multicentric validation studies adopting standardized pre-analytical and analytical workflows will be essential to improve reproducibility and facilitate the translation of urinary EV research into precision nephrology.

## 5. Urinary Transcriptome Profile in Kidney Transplant Monitoring

The development of high-throughput technologies has been one of the most significant advancements of recent years. Among the various approaches, urine transcriptome profiling emerged as a powerful tool for non-invasive diagnostic procedures and precision medicine approaches, evolving rapidly from Reverse Transcription Polymerase Chain Reaction (RT-PCR) techniques to single cell analysis. These strategies can provide specific RNA signatures of both coding and non-coding transcript derived from different cell types, such as renal cells or immune cells, reshaping kidney transplant monitoring and graft function assessment [68]. MicroRNAs (miRNAs) have attracted considerable attention because of their stability and tissue specificity, and different platforms have been developed for their quantitative and qualitative analysis [69]. Referring to urinary miRNAs, these transcripts may originate from kidney-derived cells, infiltrating leukocytes, or EVs and can, therefore, reflect multiple aspects of allograft physiology and pathology [70,71]. The miRNA profiling of urine samples derived from transplant patients has been extensively analyzed over the years. Several studies demonstrated that the quantification of urinary RNAs and differences in their expression of miRNAs could be used to detect acute kidney rejection, DGF, and allograft dysfunction [72,73]. In particular, early transcriptomic studies identified specific urinary miRNAs associated with acute TCMR, including miR-10a/10b and miR-210, with miR-210 showing strong discriminatory capacity between rejection and stable graft function and correlating with a subsequent decline in graft performance [13].

When comparing kidney transplant recipients with normal graft function to patients with DGF, Khalid and coworkers found a panel of microRNAs that were predictive of DGF onset, including miR-21. The increased expression of miR-21 has been already observed in the urine and plasma of patients with different types of AKI, highlighting its role in ischemic injury, apoptosis and fibrosis pathways and in predicting kidney transplant outcome [14,74]. Subsequent work confirmed that urinary exosomal miRNA signatures can improve diagnostic accuracy for AR compared with total urinary RNA, supporting the relevance of vesicle-associated miRNAs as more stable and kidney-enriched biomarkers [15]. More recently, multigene urinary miRNA panels derived from EVs have shown promise capacity in identifying subclinical TCMR with clinically relevant diagnostic performance. The microarray profiling revealed the presence of 38 urinary exosomal miRNAs, with three resulting upregulated and five resulting downregulated in patients with sc-TCMR [16].

Other non-coding RNAs, such as circular RNAs (circRNAs), have been detected in urine both free and carried by uEVs. CircRNAs are covalently closed molecules known for their high stability and resistance to degradation. These characteristics make their quantitative and qualitative evaluation a potential biomarker of kidney alterations. Studies on kidney transplant recipients revealed different circRNA profiles among patients with normal kidney function and patients with graft disorders. More in detail, Kölling and colleagues detected 5199 circRNAs by using microarray tools, and 363 of these molecules were differently expressed in the presence of T cell-mediated rejection. In particular, the concentration of the circRNA identified as hsa_circ_0001334 was significantly higher in the presence of rejection [17,75]. Despite being preliminary and obtained from a monocentric study, these results provide a potential non-invasive molecular signal for the monitoring of TCMR. Urinary long non-coding RNAs (lncRNAs) are commonly detected within uEV. As other non-coding RNA classes, lncRNAs are intensely studied as potential markers of pathological processes, since they are characterized by high stability in body fluids and cell-type specificity [76].

Lorenzen and colleagues performed a global urinary lncRNA expression analysis in a single-center study on a cohort of 62 transplant patients with AR and 31 subjects with no sign of rejection. The results revealed that the lncRNAs RP11-395P13.3-001 and RP11-354P17.15-001 were upregulated in patients undergoing graft rejection compared with controls. Interestingly, RP11-354P17.15-001 was associated with a higher decline in glomerular filtration rate 1 year after transplantation; also, its levels were normalized after successful antirejection therapy [18]. The robustness of the results is limited by the lack of molecular mechanisms responsible for the lncRNA release and the absence of lncRNA in situ hybridization in kidney biopsies. Although their role in regulating events such as inflammation, immune response and fibrosis is well known, urinary lncRNA profiling remains less investigated compared with other classes of non-coding RNAs. Major limitations in the analysis of lncRNA are the high variability of sample processing protocols and detection methods as well as the low abundance of this class of RNA in urine [77].

Among the different transcriptomic biomarkers, urinary messenger RNAs (mRNAs) have been the most extensively investigated in kidney transplantation. They reflect the activation of immune and inflammatory pathways occurring within the graft and can be detected non-invasively from urinary exfoliated cells. Urinary mRNAs currently represent the transcriptomic biomarkers with the highest level of clinical validation in kidney transplantation. Early studies demonstrated that urinary cell mRNA expression closely reflects intrarenal immune activation, establishing the basis for molecular monitoring through liquid biopsy [19]. A major milestone was achieved by Suthanthiran et al. in the multicenter Clinical Trials in Organ Transplantation (CTOT-04) study, which identified a three-gene urinary-cell mRNA signature (CD3ε, CXCL10/IP-10 and 18S rRNA) capable of diagnosing acute cellular rejection with high sensitivity and specificity several days before biopsy confirmation. The results revealed a shared genetic profile among urine and kidney biopsies, with a similar gene expression pattern in patients with TCMR and AMR, highlighting the diagnostic potential of urine mRNA and the possibility to combine the tissue and liquid biopsies analysis for increased accuracy of transplant monitoring. Importantly, this molecular signature was prospectively validated across multiple transplant centers, representing one of the first successful examples of clinical validation of a urinary transcriptomic biomarker panel in kidney transplantation [20,78]. More recently, the development of multigene urinary mRNA panels has improved the discrimination between stable graft function, AR, and other forms of allograft injury, highlighting the potential of urinary transcriptomics as a surrogate marker of ongoing molecular processes within the transplanted kidney [79].

Although several urinary mRNA signatures have undergone multicenter clinical validation, further assay standardization and prospective validation studies are still required before their widespread implementation in clinical decision making. The clinical implementation of urinary transcriptomic biomarkers also depends on the availability of robust and standardized sample-processing procedures. For what concerns the management of urine samples prior to analysis, filtration-based protocols have emerged as a suitable alternative to the centrifugation-based protocol. These protocols allow the collection and lysis of urinary cells using a proper filter able to retain cells and a lysis buffer that is pushed through the filter [80]. The filtration-based protocol allows patients to perform the initial urine processing steps at home, ensuring comparable purity and integrity of extracted RNA compared with centrifugation-based protocols [81].

Despite the considerable progress achieved over the past decade, several methodological limitations still prevent widespread clinical implementation of urinary transcriptomic biomarkers. RNA yield and integrity are strongly influenced by urine collection, storage conditions, processing protocols, and the relative contribution of different urinary cellular populations. Moreover, variability introduced by RNA extraction methods, sequencing platforms, normalization strategies, and bioinformatic pipelines substantially affects reproducibility across studies [82,83]. Another important limitation concerns cohort heterogeneity. Most available studies include relatively small patient populations from single transplant centers and frequently evaluate different clinical endpoints, preventing direct comparison among published datasets. External validation remains limited, and prospective multicenter studies using standardized protocols are still required before urinary transcriptomic signatures can be routinely incorporated into clinical decision making. Table 2 summarizes of the diagnostic performance of the main proposed biomarkers.

## 6. Integrated Multi-Omic Approaches and Future Perspectives

The complexity and heterogeneity of graft injuries and dysfunctions, as well as the several repair and regenerative pathways involved, require integrated molecular approaches capable of simultaneously interrogating multiple biological layers rather than relying on individual biomarkers. Therefore, multi-omic approaches have been increasingly applied to integrate information derived from transcriptomics, proteomics, metabolomics, extracellular vesicles, urinary cellular phenotypes, and clinical data, providing a systems-level understanding of graft biology [84]. Indeed, exfoliated kidney cells mainly mirror cell turnover, tissue injury and repair mechanisms, and proliferative and differentiative activities, while EVs provide information regarding intercellular communication, tissue microenvironment and molecular cargo composition. Lastly, transcriptomic analyses capture gene-expression programs associated with graft dysfunction, inflammation, immune activation, and rejection, whereas proteomic analyses characterize the functional protein landscape of the graft. Metabolomics add a complementary functional layer by measuring the downstream products of cellular metabolism, reflecting mitochondrial function, oxidative stress, amino acid metabolism, lipid remodeling, and energy homeostasis. Together, these complementary omic layers provide a substantially more comprehensive characterization of graft biology than any individual biomarker alone [85]. The development of high-throughput sequencing technologies, mass spectrometry-based proteomics and metabolomics, together with single-cell sequencing, spatial transcriptomics and high-dimensional flow cytometry, has significantly improved the integration of cellular and molecular information. These technological advances have fostered the implementation of multi-omics and systems biology approaches, enabling the characterization of complex molecular networks involved in kidney injury, tissue repair, immune activation and chronic graft dysfunction. Importantly, genuine multi-omic integration does not simply consist of analyzing multiple molecular datasets independently; instead, it requires the computational integration of complementary datasets obtained from the same patient or biological sample to identify coordinated biological pathways associated with specific clinical phenotypes [21,86]. One of the most representative examples of this approach was recently provided by Tinel et al., who integrated six high-dimensional omic datasets comprising approximately 170,000 molecular variables from 131 kidney transplant recipients and derived from blood, urine, and kidney allograft biopsies using Multi-Omics Factor Analysis (MOFA). The datasets included blood miRNA expression, blood mRNA expression assessed using different transcriptomic platforms, biopsy miRNA and mRNA expression, and urinary gene-expression data, thus enabling the joint analysis of molecular information across different biological compartments. MOFA identified eight latent factors capturing shared and compartment-specific sources of biological variability, some of which were associated with allograft rejection and reflected coordinated immune and molecular processes. Rather than identifying isolated biomarkers, the authors reconstructed latent molecular factors shared across different biological compartments, demonstrating that joint computational analysis can reveal coordinated molecular patterns that would not be captured by independent analysis of individual datasets. This work illustrates how computational integration substantially improves the biological interpretation of transplant pathology compared with conventional single-omic analyses and represents a paradigm for precision medicine in kidney transplantation [21]. Verma et al. demonstrated a remarkable concordance between urinary-cell transcriptomes and kidney biopsy gene expression profiles, showing that urinary liquid biopsy accurately reflects ongoing intrarenal molecular processes associated with acute rejection and supporting its use as a non-invasive surrogate of tissue molecular activity. However, this type of cross-compartment comparison should be distinguished from genuine multi-omic integration, since concordance between independently analyzed datasets does not necessarily imply their joint computational integration at the patient level [68]. Similarly, Burrello and colleagues integrated urinary and circulating extracellular vesicle phenotyping with longitudinal clinical follow-up, identifying EV signatures predictive of graft functional recovery after transplantation and highlighting the complementary contribution of different biological compartments to transplant monitoring. Although such approaches demonstrate the value of combining information from different biological compartments, they differ conceptually from studies such as that of Tinel et al., in which multiple omic layers are jointly integrated within the same analytical model [8].

Metabolomics has also emerged as a valuable component of integrated transplant phenotyping. Unlike transcriptomics and proteomics, which characterize gene expression and protein abundance, metabolomics reflects the functional biochemical consequences of cellular activity. High-resolution mass spectrometry and nuclear magnetic resonance (NMR)-based studies have identified alterations in amino acid metabolism, tricarboxylic acid cycle intermediates, lipid metabolism and oxidative stress in patients with acute rejection, delayed graft function and chronic allograft dysfunction [22]. Importantly, Suhre et al. demonstrated that urinary metabolite signatures associated with acute rejection are strongly influenced by graft function itself, emphasizing that metabolomic biomarkers should be interpreted together with transcriptomic, proteomic and clinical data rather than in isolation [23].

The integration of transcriptomic, proteomic, metabolomic, and clinical data gives a better understanding of disease-associated pathways and clinically relevant biomarkers. This allows a more accurate assessment of the cellular and molecular signatures detected in urine, enhancing their potential as reliable indicators of kidney alterations and disease-associated pathophysiological processes. Also, the integration of urine-derived markers with findings obtained from advanced tissue profiling technologies such as spatial transcriptomics may pave the way for the identification of patient-specific molecular signatures, also improving result interpretation [55,56,68].

Large-scale multi-omics approaches have further increased the need for machine learning and artificial intelligence (AI) tools. Recent studies have demonstrated the ability of machine learning algorithms to integrate high-dimensional molecular, clinical, histological and imaging datasets, identifying complex biological patterns associated with graft function, rejection, fibrosis and long-term clinical outcomes. As increasingly large multicenter datasets become available, AI-based analytical frameworks are expected to facilitate patient stratification, personalized risk prediction and precision medicine approaches in kidney transplantation [5,87]. However, the high dimensionality of multi-omic datasets relative to the generally limited number of patients available in transplantation studies increases the risk of model overfitting. Appropriate dimensionality reduction, feature selection, cross-validation, and independent validation are, therefore, essential when applying machine-learning approaches to these datasets.

Despite these promising advances, several challenges still limit the routine clinical implementation of multi-omic approaches. Most studies remain based on relatively small cohorts and differ substantially in sample collection, processing protocols, analytical platforms and bioinformatic pipelines, reducing reproducibility across centers. Furthermore, external validation in independent populations is still limited. Future investigations should, therefore, prioritize standardized methodologies, longitudinal sampling, multicenter collaborations, and harmonized computational workflows to facilitate the clinical translation of integrated molecular signatures [85].

**Figure 2:**
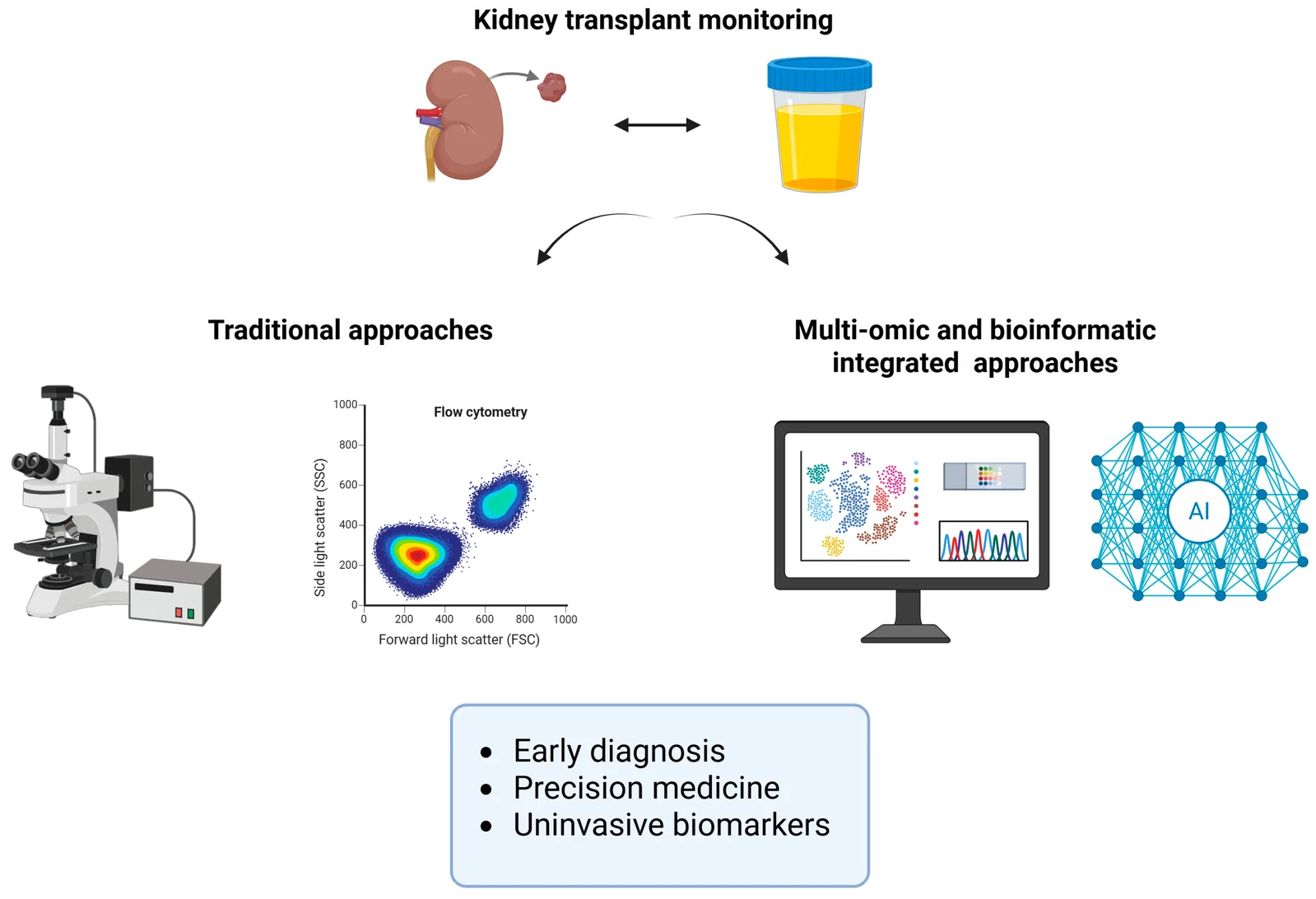
Integrated multi-omic approaches for urinary liquid biopsy analysis. Exfoliated urinary cells, extracellular vesicles, and soluble molecules can be investigated using complementary multi-omic technologies and bioinformatic tools to improve kidney transplant monitoring. Created in BioRender. Bin, S. (2026) https://BioRender.com/t4qxe88, accessed on 4 September 2026.

Overall, the integration of urinary exfoliated cells, extracellular vesicles, soluble biomarkers, transcriptomics, proteomics, metabolomics and advanced computational approaches is progressively transforming urinary liquid biopsy from a biomarker-based strategy into a systems biology platform. Rather than relying on isolated molecular markers, future diagnostic approaches will likely reconstruct patient-specific molecular networks capable of describing the biological complexity of graft injury, improving early diagnosis, predicting clinical outcomes and supporting individualized therapeutic interventions [21]. The integration of different biological sources and different analytical technologies, along with novel machine learning and AI-based tools in kidney transplant monitoring, is summarized in Figure 2.

## 7. Clinical Confounding Factors

Urinary biomarkers associated with rejection may also be altered by other clinical conditions. Indeed, several pathological processes can induce inflammatory responses, tubular injury, or changes in extracellular vesicle (EV) profiles that may overlap with the molecular signatures observed during rejection. Therefore, relevant clinical confounders should be carefully considered when assessing the specificity of a proposed biomarker and its ability to distinguish rejection from other causes of graft injury or dysfunction. In BK nephropathy, viral replication induces tubular injury and a local immune response, potentially increasing epithelial cells, mRNA/miRNA, and EVs carrying inflammatory signals similar to those observed in rejection. In 2023, Salinas et al. demonstrated that urinary cell mRNA profiles can discriminate T cell-mediated rejection (TCMR) from BK virus nephropathy (BKVN), supporting the existence of distinct molecular signatures despite the inflammatory features shared by these conditions [24].

These findings emphasize that biomarkers reflecting immune activation should be interpreted together with disease-specific molecular signals when differentiating rejection from viral injury.

In the presence of urinary tract infections and hematuria leukocytes, urothelial cells, and extracellular material originating from the urinary tract can contaminate the sample and substantially alter the cellular and EV profiles [88].

This is particularly relevant because urinary EVs participate in host–pathogen interaction, and their molecular cargo may change during infection, potentially generating inflammatory signatures unrelated to alloimmune injury. Hematuria also represents a significant technical and biological confounder in studies of urinary EV biomarkers. Experimental blood contamination of urine has been shown to progressively affect both the yield and proteomic composition of isolated urinary EVs, demonstrating that even the pre-analytical characteristics of the sample may influence biomarker measurements [89]. Recurrent glomerular disease may increase the release of glomerular- and podocyte-derived EVs and alter the protein composition of urine [90]. Therefore, some biomarkers attributed to graft injury may instead reflect glomerular injury rather than an immunological process. AKI represents an important confounder because it directly induces tubular cell loss and the release of EVs containing stress- and injury-related molecules [91].

Therefore, a biomarker based exclusively on tubular injury signals may have high sensitivity but limited specificity for rejection. Lastly, ureteral stents represent a potential confounding factor, as they may cause urothelial irritation and remodeling, inflammation, hematuria, and infections, all of which may alter the cellular and urinary EV profiles. However, the specific impact of stenting on EV biomarkers of rejection in kidney transplant recipients remains poorly studied [92].

Table 3 summarizes the main approaches in kidney transplant monitoring, their advantages and limitations. Taken together, these observations indicate that the main challenge for urinary biomarkers of rejection is not only analytical sensitivity but also biological specificity. Consequently, future validation studies should systematically account for these clinical confounders and determine whether candidate biomarkers provide diagnostic information beyond generic signals of inflammation or tissue injury.

## 8. Conclusions

Kidney transplantation requires accurate and timely monitoring strategies capable of detecting allograft injury before irreversible structural damage occurs. Although kidney biopsy remains the diagnostic reference standard, its invasiveness, limited repeatability, sampling variability, and associated risks have stimulated the search for reliable non-invasive biomarkers that can complement conventional clinical monitoring.

Among the available approaches, urinary liquid biopsy has emerged as one of the most promising strategies because it provides direct access to biological information released by the transplanted kidney. Rather than relying on a single biomarker, urinary liquid biopsy encompasses multiple complementary components—including freshly exfoliated urinary cells, soluble secretome, extracellular vesicles, and transcriptomic signatures—that reflect different aspects of graft biology, ranging from epithelial injury and immune activation to tissue repair, fibrosis, and intercellular communication.

Several urinary biomarkers have demonstrated encouraging diagnostic performance in specific clinical settings. Urinary chemokines such as CXCL9 and CXCL10, donor-derived molecular signatures, and selected urinary transcriptomic panels have shown increasing levels of clinical validation for the detection of allograft rejection and immune activation. In contrast, urinary extracellular vesicles, culture-expanded urine-derived renal epithelial cells, and integrated multi-omic signatures remain predominantly investigational tools, despite their considerable potential to improve disease phenotyping and mechanistic understanding of graft dysfunction. Importantly, these biomarkers should not be regarded as competing alternatives, but rather, as complementary sources of information that may collectively improve the non-invasive characterization of kidney allograft injury.

Despite these promising advances, important challenges still limit the clinical translation of novel urinary biomarkers. Most of the studies are monocentric, with small and heterogeneous patient populations. Differences in urine collection and processing protocols, lack of standardized analytical methodologies, variable normalization strategies, and limited external validation represent other relevant issues. Furthermore, clinically relevant confounding conditions—including urinary tract infections, BK polyomavirus nephropathy, ischemia–reperfusion injury, calcineurin inhibitor toxicity, recurrent glomerular diseases, proteinuria, and hematuria—may substantially influence urinary biomarker profiles and should be systematically considered in future investigations. These methodological limitations currently prevent the widespread implementation of most urinary biomarkers in routine transplant surveillance.

Future research should, therefore, move beyond the identification of isolated biomarkers toward the development of integrated biological models capable of combining urinary cellular phenotypes, soluble secretome, extracellular vesicles, transcriptomics, proteomics, metabolomics, and clinical information within unified computational frameworks. Large prospective multicenter studies using standardized protocols, biopsy-anchored molecular validation, and transparent artificial intelligence approaches will be essential to establish robust and reproducible biomarker signatures.

The combination of standardized multi-omic technologies with advanced computational approaches may pave the way toward precision medicine, enabling earlier diagnosis, more accurate risk stratification, individualized therapeutic decisions, and improved long-term graft survival.

## Figures and Tables

**Table 1:** Representative studies investigating urinary cellular and molecular biomarkers in kidney transplantation and their potential clinical significance.

Authors	Ref.	Urinary Material	Analytical Approach	Main Results
Pizzuti et al.	[2]	Urine-derived renal epithelial cells (URECs)	Cell phenotyping and functional characterization	URECs showed epithelial/tubular and progenitor markers.
Manonelles et al.	[3]	Urinary sediment/urinary parietal epithelial cells (uPECs)	Urinary cell phenotyping	uPECs were associated with proteinuria and chronic graft damage.
Goerlich et al.	[4]	Urinary sediment	Multiparametric flow cytometry	Rejection was associated with increased urinary T cells.
Wu et al.	[5]	Exfoliated proximal tubular cells	Immunomagnetic isolation, multispectral autofluorescence and machine learning	Tubular-cell autofluorescence differentiated major causes of graft dysfunction.
Pizzuti et al.	[6]	Cultured URECs	Cell culture and functional characterization	URECs showed immunomodulatory properties impaired by NGAL.
Knafl et al.	[7]	Urinary sediment/nephrospheres	Urinary cell culture and phenotypic assessment	Nephrospheres were associated with recovery after AKI.
Burrello et al.	[8]	Urinary and serum EVs	High-dimensional EV phenotyping	Specific urinary EV subsets were associated with renal recovery.
Jacob et al.	[9]	Urinary and plasma EVs	EV quantification and phenotyping	Urinary and circulating EV profiles differed according to rejection status.
Verdugo-Molinares et al.	[10]	Urinary EVs	EV proteomics/high-resolution mass spectrometry	uEV proteomic alterations were mainly associated with fibrosis.
Braun et al.	[11]	Small urinary EVs	EV proteomics	uEV proteomic profiles changed during transplantation.
Daniella et al.	[12]	Urinary EVs	EV proteomics	uEV protein composition was associated with pediatric rejection.
Lorenzen et al.	[13]	Urine	miRNA profiling	Urinary miR-210 discriminated TCMR from stable graft function.
Khalid et al.	[14]	Urine	miRNA profiling	A urinary miRNA panel predicted DGF.
Seo et al.	[15]	Urinary EVs	Exosomal miRNA profiling	Exosomal miRNA signatures improved acute rejection discrimination.
Tajima et al.	[16]	Urinary EVs	Exosomal miRNA profiling	Exosomal miRNAs were associated with subclinical TCMR.
Kölling et al.	[17]	Urine	circRNA transcriptomics/microarray	Specific urinary circRNAs were differentially expressed in TCMR.
Lorenzen et al.	[18]	Urine	lncRNA transcriptomics	Specific urinary lncRNAs were associated with acute rejection and graft-function decline.
Salinas et al.	[19]	Urinary cells	Multigene mRNA transcriptomics	A multigene urinary-cell signature identified acute rejection.
Suthanthiran et al.—CTOT-04	[20]	Urinary cells	mRNA transcriptomics/RT-qPCR multigene signature	A three-gene urinary-cell signature accurately identified acute cellular rejection.
Tinel et al.	[21]	Urine, blood and kidney biopsy	Integrated multi-omics/Multi-Omics Factor Analysis (MOFA)	Multi-omic integration identified rejection-associated molecular factors.
Yozgat et al.	[22]	Urine	Untargeted metabolomics	Urinary metabolic pathways differed according to graft dysfunction.
Suhre et al.	[23]	Urine	Metabolomics	Rejection-associated metabolites were strongly influenced by graft function.
Salinas et al.	[24]	Urinary cells	mRNA transcriptomics/multigene profiling	Urinary mRNA profiles differentiated TCMR from BKVN.

**Abbreviations:** AKI, acute kidney injury; AMR, antibody-mediated rejection; ATN, acute tubular necrosis; BKVN, BK virus nephropathy; DGF, delayed graft function; EVs, extracellular vesicles; IFTA, interstitial fibrosis and tubular atrophy; NGAL, neutrophil gelatinase-associated lipocalin; TCMR, T cell-mediated rejection; URECs, urine-derived renal epithelial cells; uEVs, urinary extracellular vesicles; uPECs, urinary parietal epithelial cells.

**Table 2:** Summary of the diagnostic performance of the proposed biomarkers.

Study	Ref.	Biomarker/Approach	Outcome/Rejection Phenotype	Cohort	Diagnostic Performance	Validation
**Goerlich et al., 2020**	[4]	Urinary T cells + TEC/PDX+ cells; flow cytometry	TCMR and ABMR vs. no rejection; TCMR and ABMR not discriminated from each other	63 KTRs; 39 underwent biopsy for graft dysfunction	Combined ratios: AUC 0.89–0.91; sensitivity/specificity NR	Single center; no independent external validation
**Wu et al., 2025**	[5]	Exfoliated proximal tubular-cell multispectral autofluorescence + ML	Graft rejection vs. ATN vs. non-rejection IFTA; rejection subtype not separately reported	30 KTRs: 10 rejection, 10 ATN, 10 IFTA	AutoGluon AUC: 0.95 (ATN vs. rejection), 0.92 (ATN vs. IFTA), 0.91 (rejection vs. IFTA)	Five-fold cross-validation; no external cohort
**Daniella et al., 2026**	[12]	Urinary EV proteomics (LC/MS)	Acute rejection: TCMR ≥ grade 1A, including mixed TCMR + ABMR; chronic rejection analyzed separately	29 pediatric KTRs: 7 AR, 9 chronic rejection, 13 stable; 3 healthy controls	Exploratory proteomic signature; sensitivity, specificity and AUC NR	Small single-center pilot; no external validation
**Lorenzen et al., 2011**	[13]	Urinary miR-210	Acute TCMR; predominantly subclinical; Banff IA–IIA/other grades	81 KTRs: 62 TCMR, 19 stable; 13 additional UTI controls	AUC 0.70 (95% CI 0.50–0.80); sensitivity 74%; specificity 52%	Single-center validation cohort; no external multicenter validation
**Seo et al., 2023**	[15]	3-exosomal-miRNA signature (miR-21-5p, miR-31-5p, miR-4532)	AR comprising TCMR, active ABMR and chronic active ABMR	Discovery *n* = 108; independent validation *n* = 260 (100 AR, 80 stable, 80 other graft injuries)	Discovery AUC 0.85 (95% CI 0.78–0.92). Validation: AUC 0.77 (0.70–0.84), sensitivity 99% (95–100), specificity 26% (17–38)	Independent multicenter validation cohort
**Tajima et al., 2026**	[16]	Urinary exosomal miR-7975	Subclinical TCMR; borderline changes analyzed separately	Discovery *n* = 6; validation *n* = 70 KTRs (55 samples at 3 mo; 48 at 12 mo)	miR-7975 AUC 0.825 (95% CI 0.698–0.953); in sc-TCMR with IFTA: AUC 0.858 (0.740–0.976), sensitivity 63.6%, specificity 95.6%	Single-center validation cohort; no external validation
**Kölling et al., 2019**	[17]	Urinary hsa_circ_0001334	Acute TCMR, including subclinical rejection	62 TCMR; 18 stable controls; 13 UTI controls; 10 post-treatment samples	AUC 0.85; sensitivity 70.1% (95% CI 59.4–79.5); specificity 92.3% (64.0–99.8)	Single-center cohort; no independent external validation
**Lorenzen et al., 2015**	[18]	Urinary lncRNA RP11-354P17.15-001	Acute TCMR; 51 subclinical and 11 clinically overt rejection cases	93 KTRs: 62 TCMR, 31 no rejection; 10 post-treatment samples	AUC 0.76; sensitivity 49%; specificity 95%	Single-center cohort; no independent external validation
**Suthanthiran et al., 2013 (CTOT-04)**	[20]	3-gene urinary-cell signature: CD3ε, CXCL10/IP-10, 18S rRNA	Acute TCMR (Banff IA or higher); also compared with ABMR and borderline rejection	485 KTRs; 4300 urine samples; multicenter prospective study	Primary AUC 0.85 (95% CI 0.78–0.91); external validation AUC 0.74 (0.61–0.86). TCMR vs. ABMR/borderline: AUC 0.78 (0.68–0.89), sensitivity 79% (67–91), specificity 71% (55–87)	Prospective multicenter study with external validation dataset
**Salinas et al., 2024**	[19]	CTOT-04 3-gene urinary-cell signature	TCMR, ABMR and mixed rejection analyzed separately	145 biopsy-matched urines from 126 KTRs: TCMR 47, ABMR 28, mixed 20, no rejection 50	AUC: TCMR 0.749 (95% CI 0.638–0.840); ABMR 0.780 (0.656–0.878); mixed 0.857 (0.727–0.947); any AR 0.780 (0.700–0.846)	Bootstrap optimism correction; no independent external cohort
**Salinas et al., 2023**	[24]	Portable WCHP urinary-cell 3-gene TCMR signature; BKV-VP1 mRNA	TCMR vs. no TCMR/no BKVN; BKVN assessed separately	45 KTRs; 12 TCMR biopsies from 11 patients; 29 no TCMR/no BKVN; 7 BKVN	TCMR: AUC 0.84 (95% CI 0.69–0.98), sensitivity 67% (35–89), specificity 86% (67–95). BKVN: AUC 1.00, sensitivity 86%, specificity 100%	Uses previously validated CTOT-04 cutpoint; no independent external validation of WCHP cohort

**Abbreviations:** ABMR, antibody-mediated rejection; AR, acute rejection; ATN, acute tubular necrosis; BKVN, BK virus nephropathy; IFTA, interstitial fibrosis and tubular atrophy; KTRs, kidney transplant recipients; ML, machine learning; NR, not reported; PDX, podocalyxin; sc-TCMR, subclinical T cell-mediated rejection; TCMR, T cell-mediated rejection; TEC, tubular epithelial cell; UTI, urinary tract infection; WCHP, Weill Cornell Hybrid Protocol. **Note:** Performance metrics are reported only when available in the cited studies. Rejection phenotypes are kept separate whenever the original study provided Banff-based classification.

**Table 3:** Positioning of urinary liquid-biopsy approaches against current kidney-transplant monitoring tools.

Approach	What Does It Measure?	Main Advantage	Main Limitation	Most Plausible Clinical Use
**Serum creatinine/eGFR**	Overall function of the graft	Inexpensive, simple, available everywhere	Not very sensitive or specific; it often increases when the damage is already significant	Routine screening/monitoring
**Proteinuria**	Protein loss through the glomerulus	Simple and useful for monitoring glomerular damage	Not specific to rejection; may reflect numerous kidney diseases	Screening/monitoring
**Urinary CXCL9/CXCL10**	Immune activation/cell infiltration	Biologically closer to the rejection process than creatinine	It may also increase in cases of inflammation or infection; it does not always define the phenotype.	Screening, differential diagnosis, monitoring of subclinical rejection
**dd-cfDNA**	Release of DNA derived from the graft → cellular damage	Dynamic, non-invasive, relatively early	It primarily indicates injury, not necessarily autoimmunity; it may increase in other forms of damage.	Screening, prevention of rejection, monitoring
**Kidney biopsy**	Morphology + immunohistochemistry/microscopy; Banff classification (if applicable)	It remains the diagnostic standard and allows for phenotypic characterization.	Invasive, sampling error, interpretive variability, not easily reproducible	Diagnosis/confirmation and characterization of rejection
**EV** **–** **transcriptomics**	mRNA, miRNA, lncRNA, and circRNA contained in EVs	It can provide information on which cells or pathways are activated.	Standardization and validation are still insufficient	Differential diagnosis and molecular monitoring
**EV–proteomics**	Surface proteins and cargo of EVs	It can identify signals of cellular origin and specific damage.	A strong reliance on isolation, contamination, and normalization	Diagnosis/monitoring
**EV–metabolomics**	Metabolites associated with metabolism, oxidative stress, and immune metabolism	It potentially identifies a functional/metabolic state that precedes structural changes.	It is the least mature EV approach and the one that is most difficult to standardize.	Prediction/forecasting and early monitoring, especially in research
**Integrated multi-omics EV**	RNA + proteins + metabolites, ideally along with clinical data	It potentially combines cellular origin + pathway + functional state.	Bioinformatics complexity, costs, sample size, and the risk of overfitting	Differential diagnosis and precision medicine, which are currently mainly experimental

**Note:** Emerging urinary assays are intended as complementary tools rather than replacements for conventional monitoring or biopsy. Kidney biopsy remains the reference standard for definitive phenotypic classification of rejection. **Abbreviations:** dd-cfDNA, donor-derived cell-free DNA; DGF, delayed graft function; EV, extracellular vesicle; eGFR, estimated glomerular filtration rate.

## Data Availability

No new data were created or analyzed in this study. Data sharing is not applicable to this article.

## Abbreviations

The following abbreviations are used in this manuscript:

AKI	Acute kidney Injury
AI	Artificial Intelligence
AMR	Antibody-mediated rejection
ATN	Acute tubular necrosis
DGF	Delayed graft function
ESKD	End-stage kidney disease
EVs	Extracellular vesicles
IFTA	Interstitial fibrosis and tubular atrophy
lncRNA	Long non-coding RNA
miRNA	MicroRNA
MISEV	Minimal information for studies of extracellular vesicles
mRNA	Messenger RNA
NGAL	Neutrophil gelatinase-associated lipocalin
PBMCs	Peripheral blood mononuclear cells
PDX	Podocalyxin
PECs	Parietal epithelial cells
PTCs	Proximal tubular cells
RNA	Ribonucleic acid
RT-PCR	Reverse transcription polymerase chain reaction
TCMR	T cell-mediated rejection
uEVs	Urinary extracellular vesicles
uPECs	Urinary parietal epithelial cells
